# Towards Personalized Lymphodepletion: A Population Pharmacokinetic Fludarabine Model in Patients Receiving CAR T-Cell Therapy

**DOI:** 10.3390/pharmaceutics17121592

**Published:** 2025-12-10

**Authors:** Javier Varela-González-Aller, Mario Andrés Sánchez-Salinas, Iñaki Troconiz, Gloria Iacoboni, Carla Alonso-Martínez, Maria-Josep Carreras-Soler, Cecilia Carpio, Anna Farriols-Danes, María Guerra-González, Alfredo Rivas-Delgado, Lucas Rivera Sanchez, Samantha Feijoo, Carolina Valdivia, Pere Barba, Marta Miarons

**Affiliations:** 1 Pharmacy Department, Vall D’Hebron University Hospital, Pg. de la Vall d’Hebron, 119, Horta-Guinardó, 08035 Barcelona, Spain; javiervarela@iconcologia.net (J.V.-G.-A.); carla.alonso@vallhebron.cat (C.A.-M.); annamaria.farriols@vallhebron.cat (A.F.-D.); maria.guerra@vallhebron.cat (M.G.-G.); lucas.rivera@vallhebron.cat (L.R.S.); carolina.valdivia@vallhebron.cat (C.V.); mmiarons@chv.cat (M.M.); 2 Pharmacy Department, Catalan Institute of Oncology-Avinguda de la Granvia de l’Hospitalet, 199, 08908 L’Hospitalet de Llobregat, Spain; 3 Hematology Department, Vall D’Hebron University Hospital, Pg. de la Vall d’Hebron, 119, Horta-Guinardó, 08035 Barcelona, Spain; mariosanchez@vhio.net (M.A.S.-S.); giacoboni@vhio.net (G.I.); ccarpio@vhio.net (C.C.); alfredorivas@vhio.net (A.R.-D.); samanthafeijoo@vhio.net (S.F.); 4 Pharmacometrics and Systems Pharmacology Research Unit, Department of Pharmaceutical Sciences, School of Pharmacy and Nutrition, University of Navarra, Pharmacy, Campus Universitario, 31009 Pamplona, Spain; itroconiz@unav.es; 5 Pharmacy Department, Consorci Hospitalari de Vic, Carrer de Francesc Pla el Vigatà, 1, 08500 Vic, Spain

**Keywords:** pharmacokinetics, fludarabine, population pharmacokinetic model, non-Hodgkin lymphoma

## Abstract

**Background/Objectives:** Optimal fludarabine dosing in the conditioning regimen based on population pharmacokinetic analysis (popPK) can predict outcomes in patients receiving hematopoietic stem cell transplantation. To date, there is no popPK tailored for patients receiving fludarabine as part of the lymphodepleting regimen before chimeric antigen receptor (CAR) T-cell infusion. The objective of this study was to develop a PopPK model of fludarabine in patients receiving CAR T-cell therapy. **Methods:** A prospective study was conducted at a tertiary hospital, from January 2021 to July 2022. Demographic, clinical, and analytical variables were collected. Blood samples were obtained on days 1 and 3 of the lymphodepleting regimen at 1.5, 2, 7 and 24 h post-fludarabine doses, and 30 min prior to CART-cell infusion. Fludarabine levels were analyzed through an ultra-performance liquid chromatography tandem mass spectrometry assay based on liquid–liquid extraction. Population pharmacokinetic analysis modeling was performed using nonlinear mixed-effects models (NONMEM). **Results:** Fifty-six patients (59% male) with a median age of 59 years (range 23–82) received CAR T-cell therapy (38 [68%] axicabtagene ciloleucel, 18 [32%] tisagenlecleucel) for relapsed/refractory large B-cell lymphoma. A total of 348 samples were collected for model development. A three-compartment model with first-order elimination best described the data. Body size, as represented by weight (WGT) with allometric scaling, was a significant predictor of all pharmacokinetic parameters (*p* < 0.05). Estimated glomerular filtration rate (eGFR) and the CAR T-cell construct type also showed statistical significance for fludarabine clearance (CL) (*p* < 0.05). Clearance was differentiated into non-renal and renal components. Estimates of V1, V2 and V3 volumes (the apparent volume of distribution of the central, shallow and deep compartments) were 41.2, 14.5 and 10.8 L, respectively. **Conclusions:** WGT, eGFR and type of CAR-T were predictors of fludarabine pharmacokinetics. This model offers a step toward precision-guided lymphodepletion and might support individualized dosing to optimize efficacy and minimize toxicity.

## 1. Introduction

Diffuse large B-cell lymphoma (DLBCL) is the predominant histological subtype of non-Hodgkin lymphomas (NHL). Frontline treatment for DLBCL is based on chemotherapy in combination with rituximab, leading to durable remissions in 60–70% of patients [1]. However, relapsed or refractory (R/R) disease requires intensive treatment, including high-dose chemotherapy with autologous stem cell transplant consolidation and chimeric antigen receptor T-cell therapy (CAR-T) [2].

The advent of CAR-T therapy has drastically changed the prognosis of patients with R/R lymphoid malignancies. Axicabtagene ciloleucel (axi-cel), tisagenlecleucel (tisa-cel) and lisocabtagen maraleucel (liso-cel) were approved by regulatory agencies for R/R DLBCL after two or more lines of systemic therapy [3,4,5], while axi-cel and liso-cel also received approval for second-line in patients who experienced disease progression within 12 months after completing first-line immunochemotherapy.

Lymphodepleting chemotherapy (LDC) prior to CAR T-cell infusion is a key step to create a favorable environment for the expansion and persistence of CAR T cells. This impact is achieved through the depletion of T, B and natural killer (NK) cells, including regulatory T cells, and tumor microenvironment modulation by indolamine 2,3 dioxygenase decrease and costimulatory molecule increase [6]. Another important mechanism by which lymphodepletion improves the efficacy of CAR-T is by preventing the induction of a T cell immune response against the murine single chain variable fragment of the CAR. Specifically, inclusion of fludarabine in the lymphodepletion regimen was linked with increased CAR T-cell expansion and persistence, a favorable cytokine profile and improved event-free survival (EFS) in patients with NHL. This effect is associated with the reduction of host immune competition and the stimulation of proinflammatory cytokines such as interleukin-6, interferon-γ and tumor necrosis factor-α, which drive both CAR T-cell activation and cytokine-mediated toxicities [7]. Although alternative lymphodepleting agents such as bendamustine have been explored with comparable efficacy in retrospective studies, fludarabine combined with cyclophosphamide remains the standard regimen [8].

Fludarabine is a purine analogue, intravenously administered as a monophosphate prodrug (F-ara-AMP), that is very rapidly and fully converted to the circulating metabolite F-ara-A, which is distributed intracellularly [9]. Then, intracellular phosphorylation produces the active metabolite fludarabine triphosphate (F-ara-ATP), which is incorporated into the DNA and RNA, and thereby inhibits DNA/RNA synthesis. Between 40% and 60% of fludarabine is eliminated as F-Ara-A in urine over the first 24 h after dosing, supporting renal excretion as a dominant pathway for elimination [10,11].

The importance of individualizing fludarabine dosing has been demonstrated in patients undergoing hematopoietic stem cell transplantation (HSCT) [12] and has confirmed a relationship between fludarabine exposure and treatment-related mortality that is not circumscribed only to patients with renal impairment [13]. Using the model published by Langenhorst et al. [14], better clinical outcomes, such as leukemia-free survival and the cumulative incidence of relapse, have been reported in patients showing optimal AUC targets in both B-cell acute lymphoblastic leukemia and aggressive B-cell NHL populations receiving CAR T-cell therapy [15,16]. However, there are notable differences between adult patients receiving CAR-T therapy in clinical practice and the pediatric population in which the Langenhorst model was developed, including tumor burden, inflammation, a higher number of previous treatment lines, prior hematopoietic transplantation, variability in dose density and renal disfunction, highlighting the need for a novel population pharmacokinetic model that incorporates the specific characteristics of patients undergoing CAR T-cell therapy.

The aim of this study was to develop a population pharmacokinetic (PopPK) model in adult patients undergoing fludarabine-based lymphodepletion prior to CAR-T administration, in order to elucidate the pharmacokinetic profile of fludarabine in this specific patient population.

## 2. Materials and Methods

### 2.1. Study Design

We performed a prospective, observational and single-center study including adult patients (≥18 years old) with R/R large B-cell lymphoma (LBCL) undergoing fludarabine-based lymphodepletion prior to CAR-T infusion (axi-cel or tisa-cel) after two or more lines of systemic therapy in the Hematology Department of the Hospital Universitari Vall d’Hebron (HUVH), in Barcelona (Spain). The study was approved by the Clinical Research Ethics Committee of HUVH (IP17/2020-PR(AG)682/2020), and all patients signed informed consent.

#### 2.1.1. Fludarabine Dosing

All patients received a 30-minute intravenous infusion of fludarabine in accordance with the following dosing schemes: (1) For those patients receiving axi-cel therapy, the dosing regimen consisted of 30 mg/m^2^ of fludarabine phosphate in combination with 500 mg/m^2^ of cyclophosphamide on days −5, −4, and −3 prior to the CAR T-cell infusion, in accordance with the technical sheet recommendations [3]. (2) For patients scheduled to receive tisa-cel, the dosing regimen was 25 mg/m^2^ of fludarabine phosphate in combination with 250 mg/m^2^ of cyclophosphamide on days −5, −4, and −3 preceding the cell infusion [4]. As fludarabine is administered as the monophosphate (f-ara-AMP), the corresponding f-ara-A dose equivalents were obtained by multiplying the administered f-ara-AMP doses by 0.78, according to the molecular weight ratio.

Patients with impaired renal function at the onset of lymphodepleting chemotherapy received lower doses of fludarabine. According to the technical data sheet [17], the dose of fludarabine should be reduced by up to 50% for those patients with a filtration rate between 30 and 70 mL/min, with careful monitoring of hematologic parameters to assess toxicity. However, due to the lack of specific value intervals, an internal protocol was developed. Thus, the fludarabine dose was reduced by 25% for estimated glomerular filtration rate (eGFR) values calculated according to the Cockcroft–Gault formula between 45–60 mL/min and by 40% for those between 30–45 mL/min [18]. In all situations, the doses of cyclophosphamide remained unchanged.

#### 2.1.2. Subjects

The inclusion criteria were (i) adult patients (≥18 years), (ii) diagnosis of relapsed/refractory LBCL after two or more lines of systemic therapy, (iii) patients undergoing fludarabine-based lymphodepletion prior to commercially available CAR T-cell infusion (axi-cel or tisa-cel) and (iv) patients with at least one measurable serum concentration of fludarabine.

The following variables were collected: age, sex, weight, height, body surface area (BSA) and body mass index (BMI), hematological disease, diagnosis and date of diagnosis, previous HSCT and date, ECOG performance status, date of admission and discharge for CAR-T infusion. We also gathered information about lymphodepletion start date, dose of each lymphodepleting agent across the 3 days, CAR T-cell infusion date, CAR-T construct, previous lines of treatment and use of bridging therapy.

Finally, laboratory values including hemoglobin and lymphocytes, albumin, serum creatinine and LDH were also collected on days 1 and 3 of LDC and prior to CAR-T infusion. Creatinine clearance (CrCl) was estimated using the Cockcroft–Gault formula of the Spanish Society of Nephrology [19] at two time points (pre-lymphodepletion and prior to the third administration).

#### 2.1.3. Blood Sampling Collection

Outpatient sample collection times were established based on the limited sample model (LSM) developed by Monahan et al. [20] in which they determined that time points 1 h, 2 h, 7 h and 24 h post-fludarabine infusion were associated with the best balance bias/error. Upon integration of this LSM sample model into the usual practice in the hospital, sample collection to determine fludarabine serum concentrations was performed at 1.5 h, 2 h, 24 h after the start of drug administration on days 1 and 3, and 30 min prior to CAR-T infusion. An additional 7 h sample was collected if the patient remained hospitalized during lymphodepletion. Each blood sample was collected in tube for serum determinations with gelose and centrifuged at 2000× *g* for 10 min; the serum obtained was separated into two aliquots and stored in a freezer at −80 °C.

### 2.2. Analytical Determination

Liquid chromatography mass spectrometry grade acetonitrile was obtained from Fisher Scientific (Waltham, MA, USA), while ammonium acetate (5M aqueous solution) was sourced from Sigma–Aldrich (St. Louis, MO, USA). Ultra-pure water was generated using a Milli-Q water purification system (Millipore, Milford, MA, USA). Reference standards of fludarabine and 2-chloroadenosine were procured from Toronto Research Chemicals, Inc. (North York, ON, Canada).

Quantification of fludarabine concentrations was determined in two steps. A calibration curves (CS) and quality control (QC) were initially performed by adding 5 µL of 7 different concentrations of fludarabine to 95 µL of serum to achieve final concentrations of 1, 2.5, 5, 50, 250, 500, and 1000 ng/mL for CS and 2.5, 250, and 1000 ng/mL for Low QC, Medium QC and High QC respectively. An additional blank sample lacking fludarabine was also prepared as control. For the sample preparation, in the second step of the process, fludarabine extraction from human serum was performed by mixing 100 µL of each serum, QC or CS sample with 200 µL of an internal standard solution (10 ng/mL 2-chloroadenosine in methanol at −20 °C). The mixture was vortexed, and 90 µL of supernatant was transferred to a new microtube and diluted with 90 µL of solution (1 mM ammonium acetate solution/acetonitrile, 95/5, *v*/*v*). Samples were vortexed briefly and kept at 4 °C until LC–MS/MS using a Waters Acquity UPLC system coupled with a Xevo TQ MS detector (Waters Corporation, Milford, MA, USA).

The chromatographic analyses were carried out using a Waters Acquity UPLC system coupled to a Xevo TQ MS detector. Chromatographic separation was achieved by injecting 5 µL of each sample (QC or CS) onto an Acquity UPLC HSS C18 column (Waters Corporation, Milford, MA, USA) (2.1 × 100 mm, 1.8 µm particle size) maintained at 40 °C. The mobile phase consisted of 1 mM ammonium acetate in water (mobile phase A) and acetonitrile (mobile phase B), using a gradient elution program as follows: 0–1.0 min, 5% B; 1.0–3.0 min, linear increase to 30% B; 3.0–3.5 min, increase to 90% B; 3.5–4.5 min, hold at 90% B; followed by re-equilibration to 5% B at 5.0 min, with a total runtime of 5.6 min. The flow rate was set at 0.3 mL/min. The mass spectrometer was operated in positive electrospray ionization (ESI) mode with multiple reaction monitoring (MRM) using mass transitions of *m*/*z* 286.10 > 154.00 for fludarabine and *m*/*z* 302.00 > 170.00 for the internal standard. Carry-over was excluded by performing blank injections after the upper calibrator, and matrix effects were controlled using matrix-matched calibration together with internal standard normalization. Linearity, accuracy, and precision were assessed following EMA/ICH M10 guidance [21]. The calibration curve (1–1000 ng/mL) exhibited excellent linearity (R^2^ = 0.999). Intra-day (within-batch) precision ranged from 14.3 to 18.5% CV at the lower limit of quantification (LLOQ; 1 ng/mL), with accuracy values (RE %) between −0.7 and 13.4%. For quality control samples (2.5–1000 ng/mL), precision ranged from 2.8 to 9.6% CV, and accuracy from −11.9 to 12.8%. Inter-day was 18.2% CV and accuracy 4.2% RE at the LLOQ. Across all QC levels, inter-day precision ranged from 6.8 to 8.0% CV, and accuracy from −5.3 to 6.9%, confirming assay reproducibility within EMA acceptance criteria. Data processing was performed using MassLynx software version 4.2 SCN 1014 (Waters Corporation, Milford, MA, USA), and fludarabine concentrations in serum were determined through calibration curves generated by linear regression with a 1/x weighting factor, based on the peak area ratios of the analyte to the internal standard.

### 2.3. Population Pharmacokinetics Analysis

Non-linear mixed effects modeling using the first-order conditional estimation method with INTERACTION (FOCEI) was used to develop the PopPK model using NONMEM^®^ version 7.4.3 (Icon Development Solutions, Ellicott City, MD, USA) [22]. The processing of the results, goodness-of-fit plots (GOF) and evaluation of the selected model were performed using Perl Speaks NONMEM (PsN^®^) v.5.3.0 [23], and R v4.2.3 [24]. Piraña v11.1 [25] was used to generate summary tables and reports.

Interindividual variability (IIV) was modeled exponentially assuming a log-normal distribution for each pharmacokinetic parameter associated to IIV. Data were logarithmically transformed for analysis. Selection between model candidates was made based on the following criteria: (i) comparison of the minimum objective function value, approximately equal to −2∙log(likelihood) (−2LL), assuming that for two nested models differing in one parameter, a reduction in −2LL of 3.84 or 6.61 points corresponds to significance levels of 5 and 1%, respectively, and (ii) precision of parameter estimates as quantified by the relative standard errors (RSE(%)), calculated as the ratio multiplied by 100 between the standard error and the point estimate of the parameter.

Model building followed a three-step process. First, a base population model providing an adequate description of the data was developed. Then, the impact of covariates on model parameters was investigated. Finally, the selected model was evaluated through simulation-based diagnostics.

#### 2.3.1. Base Population Model

Disposition of fludarabine in serum was described with compartmental models parameterized in apparent volumes of distribution, distribution (inter-compartmental) clearance, and elimination clearance. The possibility of non-linear pharmacokinetics in both distribution and elimination processes was considered and tested for significance. Results from model diagnostics revealed whether model assumptions were supported by the data. The presence of the non-diagonal elements of the variance–covariance matrix and the inter-occasion variability were also explored at this stage of model building. The residual model structure was selected among the additive, proportional, and combined error models in logarithmic scale.

#### 2.3.2. Covariate Selection

Selection of covariates was performed using the stepwise covariate model building method [22] with levels of significance of 5% and 1% for the forward selection and backward deletion of covariates, respectively, taking also into account the physiological plausibility and sufficient representation in the study population.

Time-varying covariates were first individually normalized by their respective median values. Continuous covariates were included in the population model, normalized by the median value of the study population. Categorical covariates were tested as proportional changes with respect to the reference category. Body weight (WGT) effects were explored using power relationships with both estimated and fixed allometric [26] exponents.

### 2.4. Model Evaluation

The simulation-based model diagnostics visual prediction-corrected Visual Predictive Check (pcVPC) [27,28] was used to perform the model evaluation. One thousand datasets of the same characteristics as the original one were simulated using the selected model. For each dataset and time bin, the 2.5th, 50th and 97.5th percentiles were computed. Then, the 95% prediction intervals of the aforementioned percentiles were obtained and represented graphically together with raw data. Potential clinical impact of the selected covariates was explored graphically. Goodness-of-fit plots and ETA-distribution diagnostics were also generated. The potential clinical impact of the selected covariates was explored graphically.

## 3. Results

### 3.1. Patient Characteristics

A total of 60 patients were enrolled in the study, and 56 were finally included in the analysis; four patients were excluded because they did not complete the fludarabine regimen as per protocol (one due to septic shock that led to death, one due to rapid clinical progression that led to treatment discontinuation, and two due to fever that caused delay in the lymphodepletion). Among the study population, 33 (59%) patients were male, and median age was 59 years (range 23–82). Mean weight at baseline was 79.6 kg (standard deviation [SD] 12.9) with obesity (IMC > 30) documented in two patients. A total of 38 (68%) patients received axi-cel, and 18 (32%) tisa-cel. The cumulative fludarabine doses for these two groups were 90 mg/m^2^ and 75 mg/m^2^, respectively, following manufacturers’ recommendations. Dose reductions of fludarabine for impaired renal function were necessary in six patients (two and four from the axi-cel and tisa-cel groups, respectively). Patients’ baseline characteristics and study data are summarized in Table 1.

### 3.2. Samples

A total of 168 fludarabine administrations were recorded, yielding 400 blood samples. Of these, 348 were included in the model development. Samples with concentrations below the LLOQ or above the upper LOQ, where further dilution was not possible, were excluded.

The concentration–time data were distributed across 348 samples, including 99 at 1.5 h, 97 at 2 h,94 at 24 h, and 51 collected 30 min before CAR-T infusion. Only seven samples were obtained 7 h post fludarabine infusion. The median number of samples per patient was six (range [1,2,3,4,5,6,7,8,9]).

**Figure 1:**
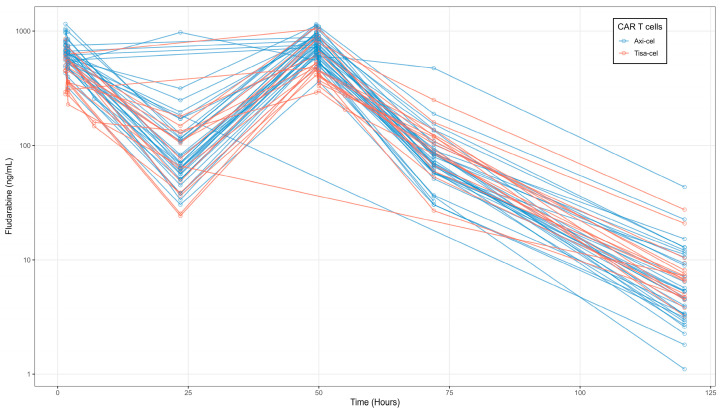
Serum fludarabine concentration–time profiles following the first intravenous injection. Open circles represent observed data; solid lines indicate linear interpolation between data points.

The overall concentration–time profiles of fludarabine are shown in Figure 1. Each open circle corresponds to an observed serum concentration, and solid lines represent the linear interpolation between sampling points.

### 3.3. Population PK Modeling

A three-compartment model best described the concentration data of fludarabine. The model was characterized in terms of volume of distribution of the central (V1), shallow peripheral (V2), and deep peripheral (V3) compartments, and elimination clearance (CL) as well as intercompartmental distribution CL between V1 and V2 (CLD2) and V1 and V3 (CLD3). This model structure provided a more accurate description of the observed serum concentration vs. time profiles, particularly during the terminal elimination phase, where simpler models exhibited significant systematic deviations.

Interindividual variability (IIV) was included on CL and V1. Shrinkage values for both random effects were low (8.9% and 18.3%, respectively), suggesting sufficient data to estimate individual parameters reliably. Analysis identified body weight (WGT), eGFR, and CAR-T construct (axi-cel vs. tisa-cel) as significant predictors of CL.

**Figure 2:**
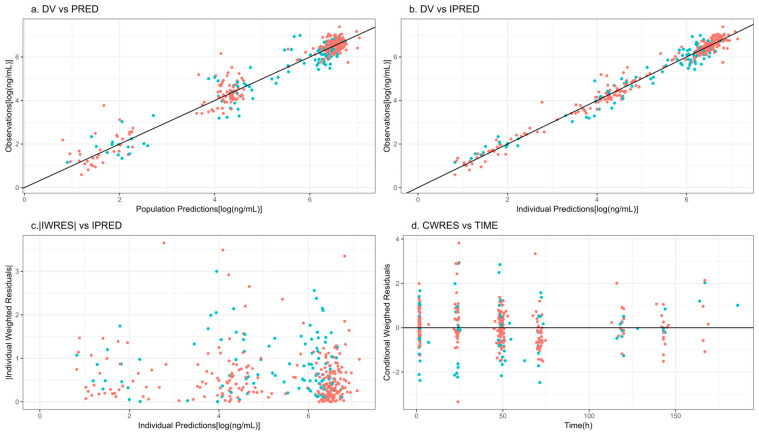
Goodness-of-fit plots. Symbols, blue: Axi-cel; red = Tisa-cel. DV, raw data; PRED, typical model predictions; IPRED, individual model predictions; |IWRES|, individual weighted residuals; CWRES, conditional weighted residuals. Solid lines show the perfect fit.

Clearance parameters for both CAR-T constructs (axi-cel and tisa-cel) along with V1 were estimated with high precision, as demonstrated by their relative standard error (RSE) values below 10%, supporting the robustness of the model fit. Interindividual variability for CL and V1 was modeled using a block covariance matrix, allowing both parameters to vary across subjects while capturing their correlation. The estimated covariance revealed a moderate positive association between CL and V1 across the population (correlation coefficient ρ ≈ 0.9). In contrast, IIV terms for intercompartmental clearances (CLD2 and CLD3) and peripheral volumes of distribution (V2 and V3) were not included in the model. This decision was based on the absence of statistical or clinical evidence supporting significant variability in these parameters, as well as the inability to estimate them with sufficient precision. Furthermore, lack of data density limited estimation of IIV on deep peripheral compartments. The estimates of the population parameters corresponding to the final model are listed in Table 2. Goodness-of-fit plots are shown in Figure 2, and ETA-distribution plots are included in the Supplementary Material. The absence of relevant trends indicates adequate model performance.

$$CL=\left[{CL}_{non-renal}+{CL}_{renal}\right]\times f(WGT)$$

$${CL}_{non-renal}\left\{\begin{array}{c} {\theta_{CAR-T}}_{1},\;axi-cel\\ {\theta_{CAR-T}}_{2},\;tisa-cel \end{array}\right.$$

$${CL}_{renal}=\theta_{CRCL}\;\times \;CRCL/96$$

f(WGT) = (WGT/70)^3/4^; g(WGT) = WGT/70; WGT, Weight.

Interindividual variability (IIV) is expressed as coefficient of variation (CV, %) calculated as $\sqrt{e^{\omega^{2}}-1}\times 100$, where ω^2^ corresponds to the variance of the random effects. The estimation of the correlation coefficient between random effects was 0.9.

**Table 2:** Final population pharmacokinetic parameter estimates.

Parameter	Description	Estimates (RSE%)	Shrinkage (%)
CL (L/h)	[*θ*_(*CAR*_*T*)_ + *θ*_*CRCL*_ × *CRCL*/96] × *f*(*WGT*)	$\theta_{CAR_{T}=1}$ = 4.4 (1.2)	-
		$\theta_{CAR_{T\;}=2}$ = 3.9 (0.9)	
		$\theta_{CRCL}$ = 1.7 (1.0)	
V1 (L)	$\theta_{V1}\times g(WGT)$	$\theta_{V1}$ = 41.2 (9.3)	
V2 (L)	$\theta_{V2}\times g(WGT)$	$\theta_{V2}$ = 14.5 (14.0)	-
V3 (L)	$\theta_{V3}\times g(WGT)$	$\theta_{V3}$ = 10.8 (3.0)	-
CLD2 (L/h)	$\theta_{CLD2}\times f(WGT)$	4.8 (5.3)	-
CLD3 (L/h)	$\theta_{CLD3}\times f(WGT)$	3.6 (0.1)	-
IIV on CL (%)	-	29.8 (25.1)	8.9
IIV on V1 (%)	-	34.8 (3.6)	18.3
Error [log(ng/mL)]	-	0.29 (18.6)	12.1

CL, total plasma clearance; V1–V3, apparent volumes of distribution of the central, shallow and deep peripheral compartments, respectively; CLD2–CLD3, inter-compartmental (distribution) clearances between the central and shallow and deep peripheral compartments, respectively; CAR_T_ = 1, Axi-cel CAR-T construct; CAR_T_ = 2, Tisa-cel CAR-T construct.

### 3.4. Model Evaluation

**Figure 3:**
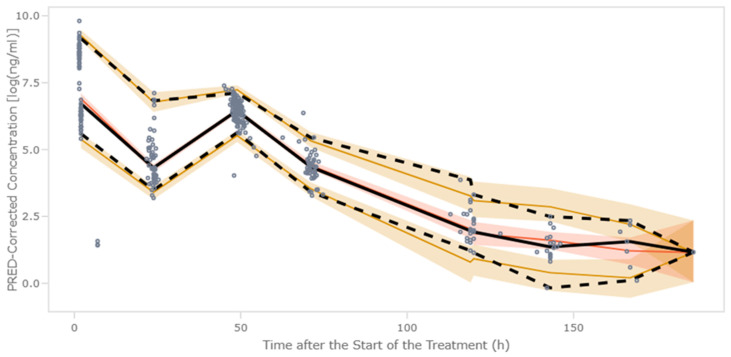
Results from the prediction (PRED)-corrected visual predictive check. Colored areas cover for each time bin, the 95% prediction intervals of the distribution of the 1000 2.5th and 97.5th percentiles calculated form the simulated dataset. Solid and dashed lines represent the median of the 1000 50th percentiles and raw data, respectively. Open circles represent PRED corrected observations.

The pcVPC showed that the median and the 2.5th and 97.5th percentiles of the observed data were in line with those from the simulation-based predictions from the model (Figure 3).

**Figure 4:**
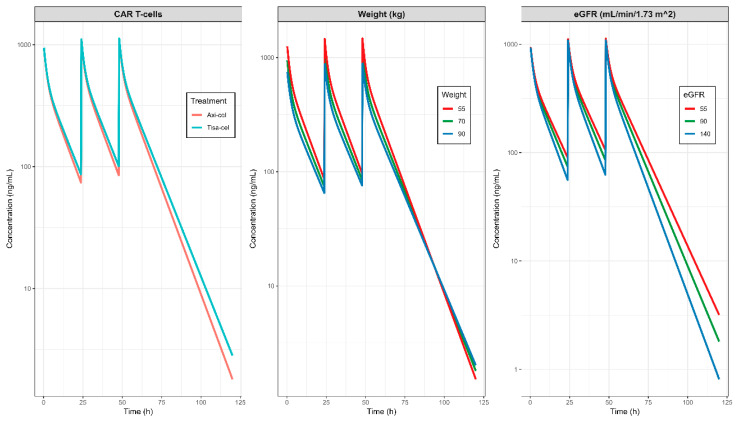
Impact of the selected covariates on the typical pharmacokinetic profiles of fludarabine. The median weight distribution was used in the left and right panels, the median eGFR in the left and middle panels, and the axi-cel CAR-T construct in the middle and right panels.

The impact of the selected covariates on the serum and the concentration of fludarabine vs. time profiles is shown in Figure 4. For each continue covariate (weight and eGFR), typical profiles were generated for the 10th, 50th, and 90th percentiles of the patient distribution. For the case of the CAR-T construct, typical profiles were generated for axi-cel and tisa-cel. The dose administered in each simulation remained constant across all scenarios and patients.

The initial simulation differentiated between the two LDC dosing regimens based on the CAR T-cell product administered, with lower concentrations observed in the treatment protocols requiring higher doses. Weight-stratified analysis showed that patients with lower weight initially achieved higher concentrations under fixed dosing, but this group exhibited the lowest terminal concentrations over time. Finally, eGFR had a marked effect on plasma concentrations, with concentration profiles diverging immediately post administration and resulting in a five-fold difference between the 10th and 90th percentiles at final simulated time points. Although this difference is consistent with reduced renal elimination, its exact magnitude should be interpreted within the context of residual variability and model accuracy. Patients with impaired eGFR presented progressive fludarabine accumulation and delayed elimination.

## 4. Discussion

In our study, we aimed to build a specific popPK model using nonlinear mixed-effects analysis to optimize fludarabine-based LDC in patients with R/R LBCL receiving CD19-targeted CAR-T therapy. Fludarabine PK was best characterized using a three-compartment model with first-order elimination. Weight, renal function and type of CAR-T construct showed statistically significant covariate effects. To the best of our knowledge, this is the first pharmacokinetic model developed exclusively in patients receiving CAR T-cell therapy, with the aim of characterizing fludarabine pharmacokinetics in this specific patient population.

Several PopPK models have characterized fludarabine disposition in HSCT populations, mainly in adults or mixed pediatric–adult cohorts [14,29,30]. Most identified body size and renal function as the main predictors of fludarabine clearance, reflecting the predominant renal elimination of F-ara-A and the strong correlation between drug clearance and creatinine clearance [11,12]. Langenhorst et al. [14] developed a three-compartment model that incorporated renal function as a major determinant of fludarabine CL. Based on the observed reduction in clearance among patients with eGFR below 120 mL/min/1.73 m^2^, the authors proposed a carboplatin-like dosing algorithm to improve individualization of therapy. In contrast, Sanghavi and Ivaturi [29,30] described the data adequately using two-compartment models, reflecting differences in patient populations and sampling design. However, these models were developed in transplant settings, typically involving younger patients with preserved renal function, and may not accurately represent the clinical profile of CAR-T recipients, who are often older, heavily pretreated and present variable renal function. Beyond these pharmacokinetic considerations, the quality and intensity of lymphodepletion itself have been shown to influence CAR T-cell expansion and clinical efficacy. Patients with aggressive NHL achieving a favorable cytokine profile after intensive conditioning demonstrated superior progression-free survival compared with those exhibiting similar depletion intensity but lacking such cytokine modulation [31]. A CAR T-specific PopPK model is therefore warranted to optimize lymphodepletion and reduce interindividual variability in this population [29,30].

Across published models, total fludarabine CL ranged from approximately 5 to 7 L/h/70 kg in adults and mixed cohorts, while Langenhorst reported 3.98 L/h/70 kg using a three-compartment model. While Sanghavi confirmed eGFR and WGT as the main clearance determinants in adults, substantial unexplained variability remained even after covariate adjustment. More recently, a three-compartment model [32] distinguished renal and non-renal clearance components of unbound fludarabine, confirming eGFR and WGT as key covariates but still exhibiting high interindividual and inter-occasion variability, suggesting additional unexplained sources of pharmacokinetic variability. For a typical 70 kg patient with a median eGFR value (96 mL/min), the predicted CL was 6.1 L/h for axi-cel and 5.6 L/h for tisa-cel. These estimates are consistent with the adult ranges reported in previously published PopPK studies.

In our model, allometric scaling based on WGT best accounted for body size differences. Subsequent inclusion of eGFR in the model eliminated any residual body size-independent effects of age. Consistent with the literature, and biologically plausible given the known extensive renal elimination of fludarabine through both glomerular filtration and active tubular secretion, the inclusion of CrCl as predictor of fludarabine CL was expected. The estimated volumes of distribution for the central, shallow peripheral, and deep peripheral compartments were 41.2, 14.5, and 10.8 L, respectively. The volume of distribution depends on plasma protein binding and total body water distribution, typically representing 60–75% of body weight [33]. Population pharmacokinetic models of fludarabine have reported values ranging from 9.6 to 39 L/70 kg for V1, 8 to 20 L/70 kg for V2, and 16 to 50 L/70 kg for V3, supporting the physiological plausibility of the estimates obtained in our model [14,32]. Finally, type of CAR-T construct as a covariable could be explained by complex, multifactorial biological differences between the patient populations treated with each product. Such differences may include variations in disease biology, prior therapies, tumor burden, and individual immunologic or metabolic profiles. Although the inclusion of the CAR-T construct led to a statistically significant reduction in the OFV, the minimal impact on the typical parameter estimates and the behavior of the diagnostic plots indicates that this effect is unlikely to be clinically meaningful and may instead reflect unmeasured covariates or other patient-specific factors. These findings highlight the importance of fludarabine dosage personalization to enhance expansion and persistence of the engineered cells and increase CAR-T efficacy.

We acknowledge that our study has certain limitations. First, the number of patients included in the analysis and the relatively short recruitment time (19 months); additionally, the limited sampling at the 7 h post-fludarabine time point may have reduced the precision of V3 and CLD3 estimates, given that this window captures the β/γ transition. The latter is due to the high number of patients who received lymphodepleting chemotherapy in the outpatient setting, making sample acquisition challenging. However, this cohort did encompass more than 340 serial samples from patients with LBCL treated with commercially available CAR T cells. In addition, although the sampling schedule was informed by an LSM originally developed in HSCT populations, this may entail minor limitations when applied to CAR-T recipients. Second, this was a single-center study, which might not fully capture the heterogeneity of clinical practice or patient populations across institutions. Third was the lack of pharmacogenetic data, specifically the non-determination of the rs2295890 mutation involved in the pharmacokinetics of fludarabine as described by Mohanan [20], associated with lower plasma concentration of F-ara-A, compared with individuals with the wild-type genotype. Although its incorporation could improve the model, its absence should not compromise its validity. Fourth, stepwise covariate selection may carry a certain degree of Type I error, potentially leading to the identification of spurious associations between covariates and pharmacokinetic parameters. While this is an inherent limitation of the method, the overall predictive performance of the model is expected to remain robust. Finally, while the current study focuses on characterizing fludarabine pharmacokinetics, we acknowledge that the absence of an exposure–response analysis (AUC vs. neurotoxicity, CRS, or CAR-T expansion) limits the immediate clinical applicability. Larger multicenter prospective studies will be warranted to confirm the predictive value of this model and its potential utility for individualized fludarabine dosing in CAR-T therapy.

The study also presents unprecedented strengths. To the best of our knowledge, this is the first population pharmacokinetic model developed prospectively for CAR-T cell patients. Notably, the fludarabine label only recommends dose reduction (up to 50%) when eGFR falls below 70 mL/min/1.73 m^2^. However, Langenhorst et al. reported decreased fludarabine CL in patients with eGFR below 120 mL/min/1.73 m^2^ [14]. Considering that chronic kidney disease (CKD) is a frequent finding in cancer patients undergoing treatment and that approximately 10–20% of these have ≥stage III CKD (eGFR < 60 mL/min/1.73 m^2^) [34], our popPK model would serve as a useful tool for precise dose individualization in this patient population. Our results allow the utilization of PopPK models to modulate lymphodepletion on an individual patient basis. Using a specific predictive model for fludarabine exposure to establish an optimal dose range has the potential to improve outcomes in this LBCL patient population, of which 40–50% do not experience long-term survival [35]. Moreover, as fludarabine-based lymphodepletion is widely used across CAR T-cell indications and products, this tailored approach could be used in a large number of patients with both malignant and non-malignant conditions [35,36]. However, real-time approaches with narrow windows for intervention such as the one proposed herein have intrinsic challenges in terms of financial and logistical barriers that potentially limit its use. Blood sampling for fludarabine levels and the model designed were developed to facilitate the integration of therapeutic drug monitoring into routine clinical practice, making dose individualization more accessible. Future studies will shed light on the applicability of the results of pharmacokinetic-based approaches in real-world practice.

## 5. Conclusions

This study developed a specific popPK model using nonlinear mixed-effects analysis for patients with R/R LBCL receiving fludarabine-based lymphodepletion prior to CAR-T therapy to optimize exposure to this purine analogue. A three-compartment model with first-order elimination best described the data. Renal function, ABW, and type of CAR-T construct were identified as statistically significant covariates. By optimizing fludarabine exposure during lymphodepletion, this model represents a step towards personalized dosing strategies that could be used as a future tool to optimize CAR T-cell efficacy.

## Figures and Tables

**Table 1:** Baseline characteristics of the patients included in the population pharmacokinetic model (*n* = 56).

Variables	Study Population *n* = 56
Male sex, *n* (%)	33 (59)
Age; median (range)	59 (23–82)
Weight (kg)	
Mean (SD)	79.6 (12.9)
Median (range)	82.5 (52–101)
Body mass index (kg/m^2^)	
Mean (SD)	25.2 (3.5)
Median (range)	25.3 (18.0–36.2)
CAR-T construct; *n* (%)	
Axi-cel	38 (68)
Tisa-cel	18 (32)
Histological diagnosis; *n* (%)	
Diffuse large B-cell lymphoma	30 (54)
Transformed from indolent lymphoma	20 (36)
High-grade B-cell lymphoma	3 (5)
Other *	3 (5)
ECOG; *n* (%)	
0	32 (57)
1	20 (36)
2	4 (7)
Prior lines of treatment; *n* (%)	
2	45 (80)
≥3	11 (20)
Prior HSCT; *n* (%)	21 (37)
Serum Albumin (g/dL)	
Median (range)	4 (2.7–4.8)
Renal Function [eGFR: mL/min/1.73 m^2^]	
Mean (range)	90 (39.0–213.9)

* Other: T cell histiocyte large B cell lymphoma, intravascular large B cell lymphoma. Abbreviations: CAR-T, Chimeric Antigen Receptor T cell; ECOG, Eastern Cooperative Oncology Group; HSCT, Hematopoietic Cell Transplantation; eGFR, estimated Glomerular Filtration Rate.

## Data Availability

The data presented in this study are available on request from the corresponding author. (The data are not publicly available due to confidentiality of patients’ private data).

## Supplementary Materials

The following supporting information can be downloaded at: https://www.mdpi.com/article/10.3390/pharmaceutics17121592/s1, Figure S1: Distribution of the ηCL (left) and ηV (right) variables.
