# Supplementary material for: Towards Personalized Lymphodepletion: A Population Pharmacokinetic Fludarabine Model in Patients Receiving CAR T-Cell Therapy
Source: Pharmaceutics. 2025 Dec 10;17(12):1592. doi: 10.3390/pharmaceutics17121592 (PMC12736365; doi:10.3390/pharmaceutics17121592)
Supplement: Supplementary file 1 [file pharmaceutics-17-01592-s001.zip › pharmaceutics-3981670-supplementary.pdf]

<u>**Supplementary data**</u>

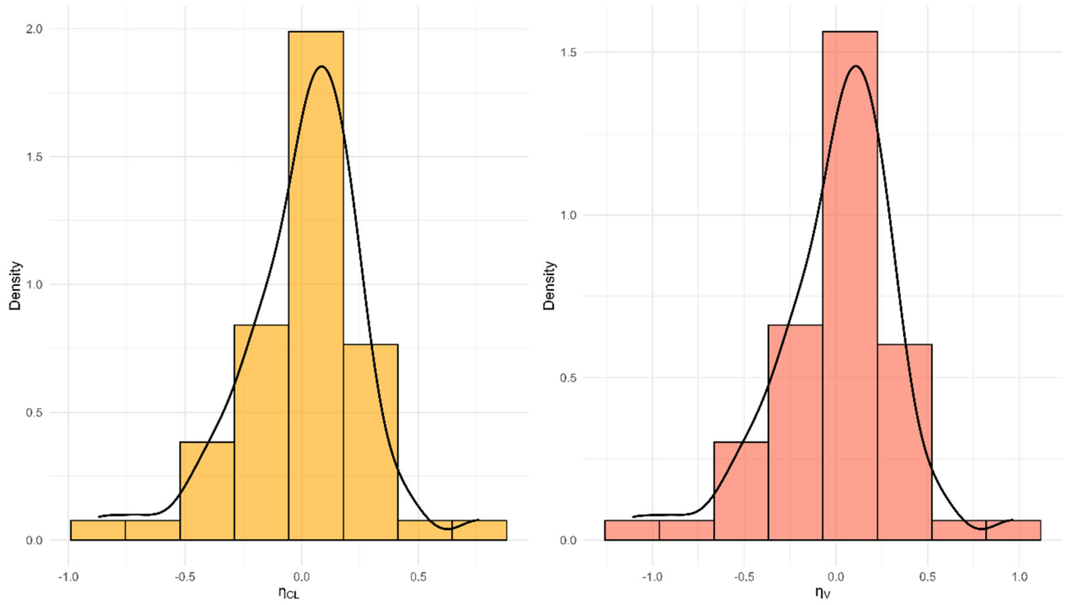

Figure S1: Distribution of the ηCL (left) and ηV (right) variables. The figure shows the histogram of the variables together with the smoothed density curve obtained by kernel density estimation. ηCL and ηV variables represent the individual random effects of the apparent volume of the central compartment and total clearance, respectively.
